# Case Report: Anti-LGI1 Encephalitis Following COVID-19 Vaccination

**DOI:** 10.3389/fimmu.2021.813487

**Published:** 2022-01-05

**Authors:** Yair Zlotnik, Avi Gadoth, Ibrahim Abu-Salameh, Anat Horev, Rosa Novoa, Gal Ifergane

**Affiliations:** ^1^ Department of Neurology, Soroka University Medical Center, Ben Gurion University of the Negev, Beer-Sheva, Israel; ^2^ Department of Neurology, Encephalitis Center, Tel-Aviv Medical Center, Tel-Aviv, Israel; ^3^ Department of Radiology, Soroka University Medical Center, Ben Gurion University of the Negev, Beer-Sheva, Israel

**Keywords:** case report, anti-LGI1 autoimmune encephalitis, COVID-19 vaccination, COVID-19 vaccination adverse effect, autoimmune encephalitis (AE)

## Abstract

Anti-leucine rich glioma inactivated 1 (LGI1) autoimmune encephalitis (AE) is characterized by cognitive impairment or rapid progressive dementia, psychiatric disorders, faciobrachial dystonic seizures (FBDS) and refractory hyponatremia. Since December 2020, millions of people worldwide have been vaccinated against COVID-19. Several soft neurological symptoms like pain, headache, dizziness, or muscle spasms are common and self-limited adverse effects after receiving the COVID-19 vaccine. However, several major neurological complications, despite the unproven causality, have been reported since the introduction of the COVID-19 vaccine. Herein, we describe a 48 years old man presenting with rapidly progressive cognitive decline and hyponatremia diagnosed with anti LGI1 AE, occurring shortly after the second dose of mRNA COVID -19 vaccine and possibly representing a severe adverse event related to the vaccination. Response to high dose steroid therapy was favorable. As millions of people worldwide are currently receiving COVID-19 vaccinations, this case should serve to increase the awareness for possible rare autoimmune reactions following this novel vaccination in general, and particularly of anti-LGI1 AE.

## Introduction

Autoimmune encephalitidies (AE) is an infrequent, newly described group of neurological inflammation diseases commonly associated to specific autoantibodies. Various subgroups of AE are distinguished by these autoantibodies, which may lead to specific clinical presentations and different prognoses. Among them, anti-leucine rich glioma inactivated 1 (LGI1) encephalitis is a treatable etiology of AE. Anti-LGI1 AE is characterized by cognitive impairment or rapid progressive dementia, psychiatric disorders, faciobrachial dystonic seizures (FBDS) and refractory hyponatremia (1–4). It is also considered as a subtype of limbic encephalitis usually occurring without any detectable paraneoplastic cause (5, 6). It is responsive to immunotherapy treatment including steroids, intravenous immunoglobulin (IVIG) and other immunosuppressive agents (7).

Autoimmune conditions such as Guillain-Barré syndrome, acute disseminated encephalomyelitis and cerebellitis can sometimes present after immunization (8–11).

During the past year, the novel coronavirus disease 2019 (COVID 19) has infected more than 100 million people worldwide. In December 2020, the first two vaccines were approved by the FDA through emergency use authorization in the United States. These vaccines are based on the mRNA vaccine platform and were developed by Pfizer/BioNTech and Moderna. Published safety and efficacy trials reported high efficacy rates of 94-95% after two interval doses, in conjunction with limited side effects and a low rate of adverse reactions (12).

Herein, we report a 48 years old man presenting with rapidly progressive cognitive decline and hyponatremia diagnosed with anti LGI1 AE, occurring shortly after the second dose of mRNA COVID -19 vaccine.

## Case Description

A 48 year old man, with no prior medical history, had started complaining of severe fatigue that developed a few days following his second dose of vaccination for COVID-19 (Pfizer mRNA vaccination). About 2.5 weeks following the vaccination, his wife started noticing memory deficits and anterograde amnesia. There were no abnormal or involuntary movements, no fever and no behavioral changes. The patient had no complaints regarding his sleep and there were no autonomic disturbances.

He was hospitalized a few days later for further evaluation. His neurological exam was normal apart from a Montreal Cognitive Assessment (MoCA) score of 18/30 (normal range 26-30), representing severe impairments in short term memory, temporal orientation, abstraction and language skills.

**Figure 1:**
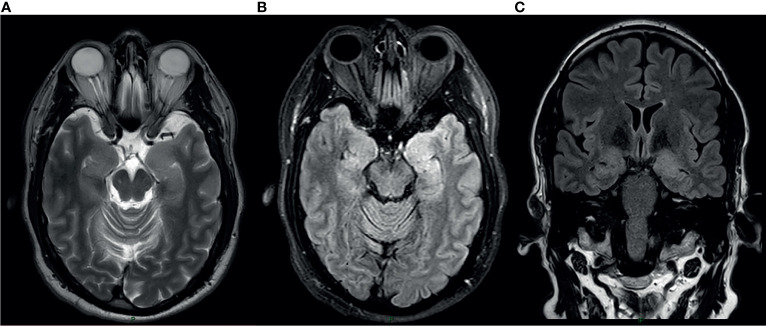
MRI at the time of presentation. Axial T2 Weighted Image **(A)**, Axial Fluid attenuated inversion recovery(FLAIR) sequence **(B)** and coronal FLAIR **(C)** show cortical thickening and hyper intense signal in the medial aspect of bilateral temporal lobes Left>Right.

Laboratory tests demonstrated serum sodium level of 132 mEq/L (normal range 135-145), tumor markers (CEA, AFP, CA125, CA19–9, CA15–3) and paraneoplastic neuronal antibodies including anti-Hu, Ri, Yo, Ma/Ta, Amphiphysin, CV2, SOX1, Tr (Euroimmun) were negative. Electroencephalogram didn’t demonstrate epileptic activity or other abnormal findings. Cranial magnetic resonance imaging (MRI) showed hyper intense signal on both medial temporal lobes (more on the left) including the parahyppocampal gyrus on T2-weighted fluid-attenuated inversion recovery and diffusion weighted imaging (**Figure 1**).

Total body computed tomography (CT scan) showed a few liver cysts and an adrenal finding consistent with adenoma, with no other signs of a solid tumor.

Serology for COVID-19 antibodies was positive (probably indicative for response to the vaccination, as the patient had not developed COVID-19 infection prior to his vaccination).

Cerebrospinal fluid analysis demonstrated normal protein and glucose without pleocytosis. CSF cultures were negative. Autoimmune cell-based encephalitis panel (Euroimmune) was positive for anti LGI1Ab both in the serum and CSF. All of the other antibodies tested were negative (anti-Neuronal NMDR, -GABAβ, - CASPR2, - AMPAR1, - AMPAR2, - Amphiphysin, - CV2, - PNMA2, -Ri, - Yo, - Hu, - Recoverin, - Soxi, - Titin).

Based on these findings the patient was diagnosed with anti-LGI1 AE and was treated initially with high dose methylprednisolone (1 gram daily for 5 consecutive days) with a good response. His MoCA score after this treatment improved to 22/30. He continued treatment with oral prednisone (1mg/kg) and his MoCA score 2 weeks later improved to 25/30 (on a different version of MoCA), representing marked improvement in temporal orientation, short term memory and language, though executive skills were impaired.

Serum sodium levels were normalized 3 weeks following his presentation.

The patient continued to improve gradually. 4 months following his presentation his MoCA score improved to 27/30, reflecting improvement in most modalities that were impaired. He still faces some executive skills difficulties.

## Discussion and Conclusions

Herein, we described a case presenting as rapidly progressive cognitive decline and mild hyponatremia, occurring after a period of about 2 weeks of severe fatigue that started days after the second dose of mRNA COVID-19 vaccination. The clinical manifestations, positive LGI1-Ab in both CSF and serum, neuroimaging findings, the treatable effect and favorable prognosis contributed to the diagnosis of anti-LGI1 AE.

Neurological symptoms like pain, headache, dizziness, or muscle spasms are common and self-limited adverse effects after receiving the COVID-19 vaccine. However, major neurological complications, despite the unproven causality, have been reported since the introduction of the COVID-19 vaccine, such as facial palsy, Guillain-Barré syndrome (GBS), seizures, strokes, transverse myelitis, acute disseminated encephalomyelitis (ADEM) and postvaccinal encephalitis (13–16).In addition, an unexplained acute encephalopathy state has also been described after receiving the COVID-19 vaccine (13, 17, 18). The pathophysiology of those complications is still not well understood and only based on hypotheses. In general, vaccinations can cause a strong expression of proinflammatory cytokines and a T cell response (16, 19, 20). After vaccination, antigens are recognized as potential pathogens by both conserved pathogen- and damage-associated molecular patterns as well as pattern-recognition receptors that are found on local or peripheral circulating immune cells (eg monocytes and macrophages) and on resident stromal cells (20). Transcription and induction of many target genes occurs, resulting in synthesis and release of pyrogenic cytokines (ie interleukin [IL]-1, IL-6, tumor necrosis factor-alpha [TNF-α], and prostaglandin-E2) into the bloodstream. These may mimic the response to natural infection. Following stimulation, the immune system initiates a complex series of innate immune events, releasing mediators and products of inflammation to the circulation that can affect other body systems to cause systemic side-effects, and ultimately can cause neuroinflammation in some subjects after microglia activation, depending on the immunogenetic background and the innate immune memory (19, 20).

Regarding mRNA vaccines, it has been proposed that spike protein expression by human cells (after translation of the mRNA COVID-19 vaccine by human cells) might trigger an inflammatory reaction, similar to that induced by the virus itself, leading to those neurological complications (17, 21). However, other mechanisms might be involved.

We hypothesize that this case of anti- LGI1 AE occurring shortly after COVID-19 vaccination, may represent a possible rare adverse event to the vaccination. We base our theory mainly on the temporal course of the symptoms, which started a few days after the vaccination. The patient received a vaccination that is meant to activate the immune system and shortly after developed an uncommon autoimmune response. He had no other febrile or medical illness prior to the disease onset.

Recently published case reports of post COVID-19 vaccine encephalitis fulfilled the criteria of possible autoimmune encephalitis, however no pathological antibody was found, as opposed to our case. Similar to our case, these patients responded to immunosuppressive therapy with corticosteroids and exhibited a relatively benign course without severe sequela (15, 16).

However, since millions of people in Israel have been vaccinated and we have not noticed a nationwide overall increase in the incidence of autoimmune encephalopathies as compared to the previous 2 years, there remains a possibility that there is no causality between the vaccination and this patient’s illness. Some cases of neurological conditions may occur within the post-vaccination window by chance alone, and as we only present a case report of a single patient, we are aware that causality cannot be proved without robust evidence from epidemiological data or animal studies. Furthermore, as this is only a single case report, we still believe that the benefits of vaccination outweigh the risks of ongoing vaccination programs.

In summary, AE is rare and its causes are still largely unknown and are only beginning to unfold. It is an under-recognized condition and has a favorable prognosis if treated promptly. Our case, which describes this condition following COVID-19 vaccination increases the awareness for possible rare autoimmune reactions following this novel vaccination in general, and particularly of anti-LGI1 AE. This awareness is crucial as early recognition and treatment is critical and necessary to achieve a favorable prognosis.

## Patient Perspective

Our patient was relieved when a diagnosis of a treatable condition was made, as he and his family were very concerned when the symptoms appeared. He noted a fast improvement in his condition a few days after steroid therapy was initiated. He gladly agreed to share his case after understanding that he suffered a rather rare condition.

## Data Availability Statement

The original contributions presented in the study are included in the article/supplementary material. Further inquiries can be directed to the corresponding author.

## Ethics Statement

Written informed consent was obtained from the individual(s) for the publication of any potentially identifiable images or data included in this article.

## Author Contributions

YZ, IA-S, AH, and GI treated the patient. RN was responsible for the acquisition and interpretation of neuroradiologic imaging. AG was responsible for the interpretation of the autoimmune encephalitis panel and consulted on the proper treatment and follow up. YZ, AG, and GI wrote the manuscript. All authors were involved in the analysis and interpretation of findings, they proved the manuscript, contributed for important intellectual content, and contributed to writing and approved the final manuscript.

## Conflict of Interest

The authors declare that the research was conducted in the absence of any commercial or financial relationships that could be construed as a potential conflict of interest.

## Publisher’s Note

All claims expressed in this article are solely those of the authors and do not necessarily represent those of their affiliated organizations, or those of the publisher, the editors and the reviewers. Any product that may be evaluated in this article, or claim that may be made by its manufacturer, is not guaranteed or endorsed by the publisher.
